# Supplementary material for: A set of constitutive promoters with graded strengths for gene expression in diverse cyanobacterial strains
Source: Appl Environ Microbiol. 2026 Jul 2;92(7):e00598-26. doi: 10.1128/aem.00598-26 (PMC13390492; doi:10.1128/aem.00598-26)
Supplement: Supplemental material — Fig. S1 to S5; Tables S1 to S4. [file aem.00598-26-s0003.docx]

Supplementary Figures

Figure S1. Initial PconII*-YFP fluorescence measurements of *S. elongatus* clones. Clones are ordered by YFP expression level from highest to lowest. The library of PconII* promoter variants was constructed by randomizing the two nucleotides in the extended -10 region and the three most conserved nucleotides (Strategy 1) or the three least conserved nucleotides (Strategy 2) in the -10 region of PconII. A total of 1643 individual clones including controls were evaluated and 341 clones were selected for further characterization.

Figure S2. Fluorescence measurements for the initial sets of library clones selected for each cyanobacterial strain. For each strain, the clones were sorted and grouped according to the PconII* expression level (YFP expression). Each group was color-coded using the final grouping in *Anabaena* so that each clone can be traced back to the original group in *S. elongatus*. Expression levels are shown as normalized YFP intensity, relative to the maximum signal (scaled × 10^5^). The black horizontal line represents the YFP expression level obtained with the original PconII promoter. Control data are not available for the *Synechocystis* experiment.


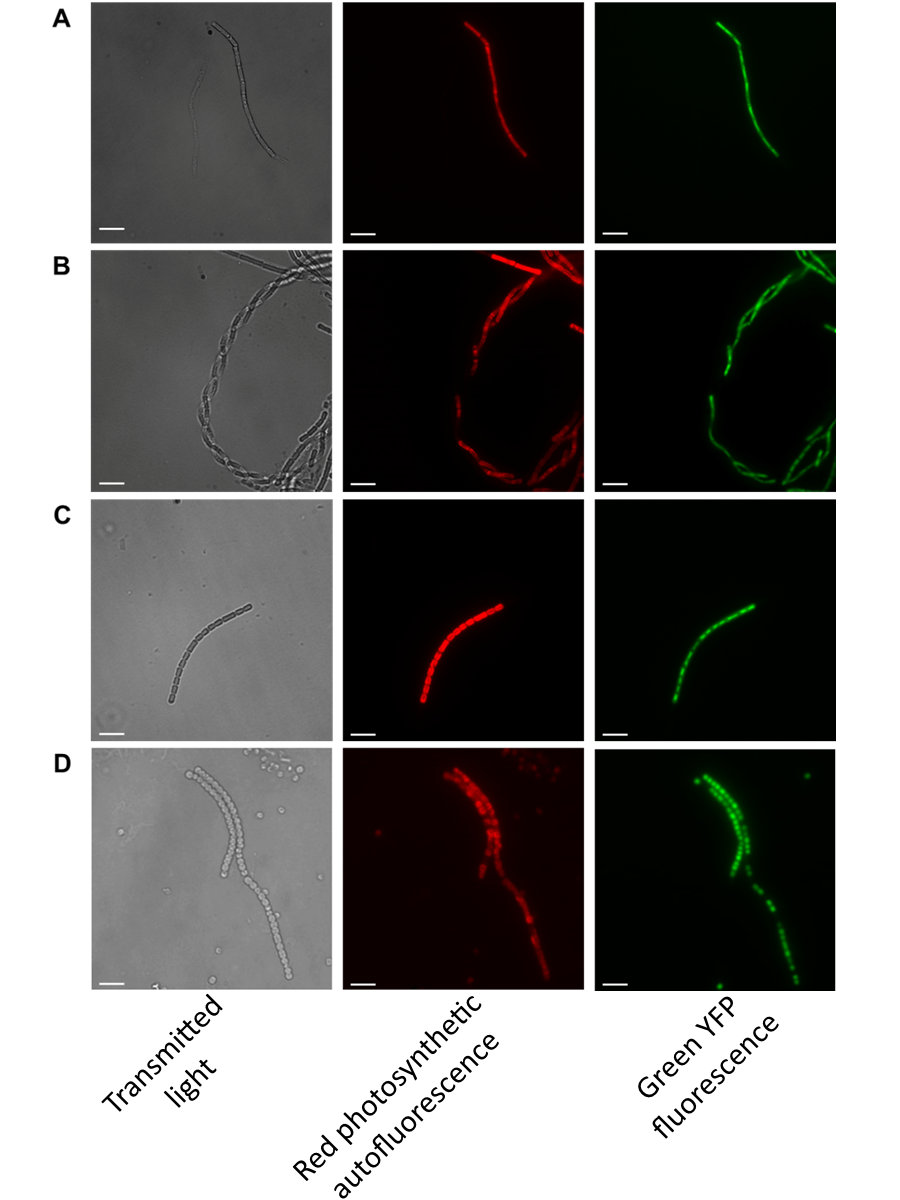


Figure S3. Micrographs of bioprospected exconjugant strains. Isolated cyanobacterial strains from Mono Lake expressing spectinomycin/streptomycin resistance and YFP fluorescence after conjugation with pAM5409. Left column, transmitted light; middle column, red autofluorescence of photosynthetic pigments; right column, green YFP fluorescence. Bioprospected strains: (A) ML2A, (B) ML2C1, (C) ML2C2, and (D) ML3B. Scale bars, 5 μm.


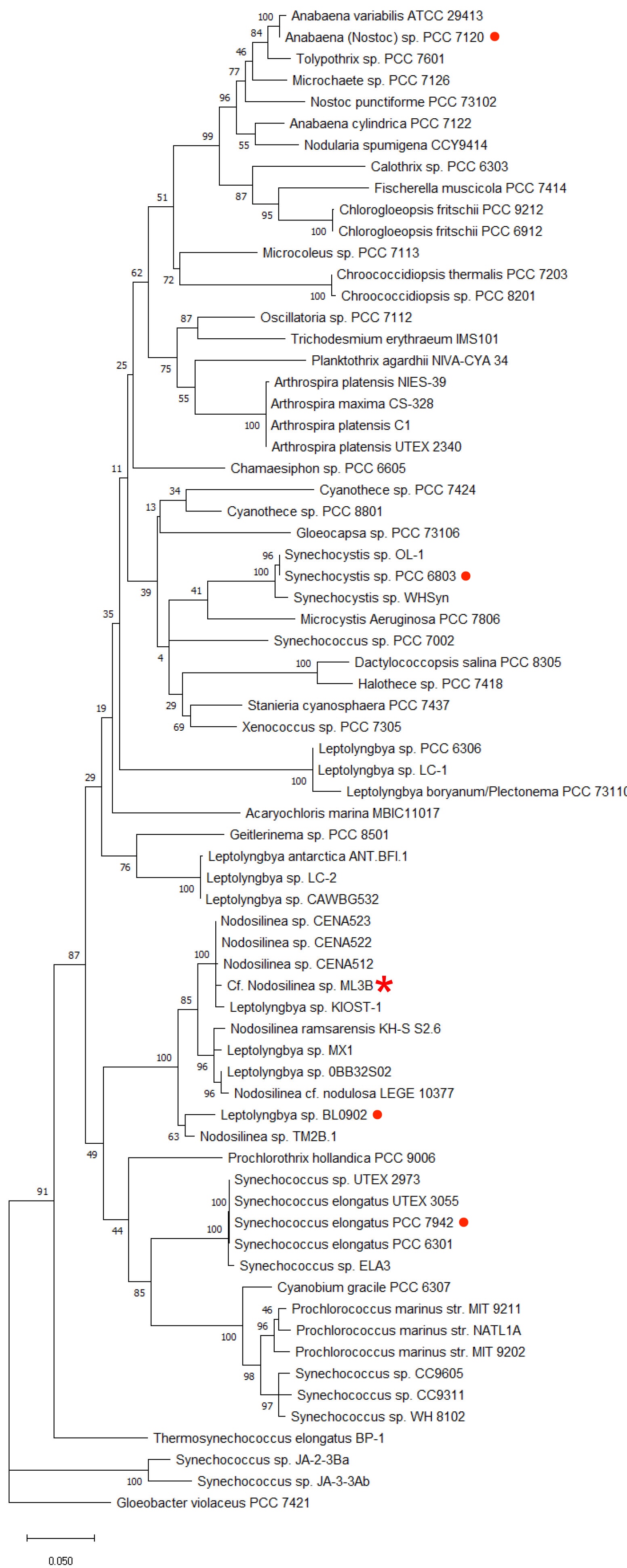


Figure S4. Phylogenetic tree based on 16S rRNA gene sequences for identification of strain ML3B. The evolutionary history was inferred by using the Maximum Likelihood method and General Time Reversible model. The tree with the highest log likelihood (-14053.35) is shown. Evolutionary analyses were conducted in MEGA X. Strain ML3B is marked with an asterisk (*) and the four standard research strains are marked with dots (•).


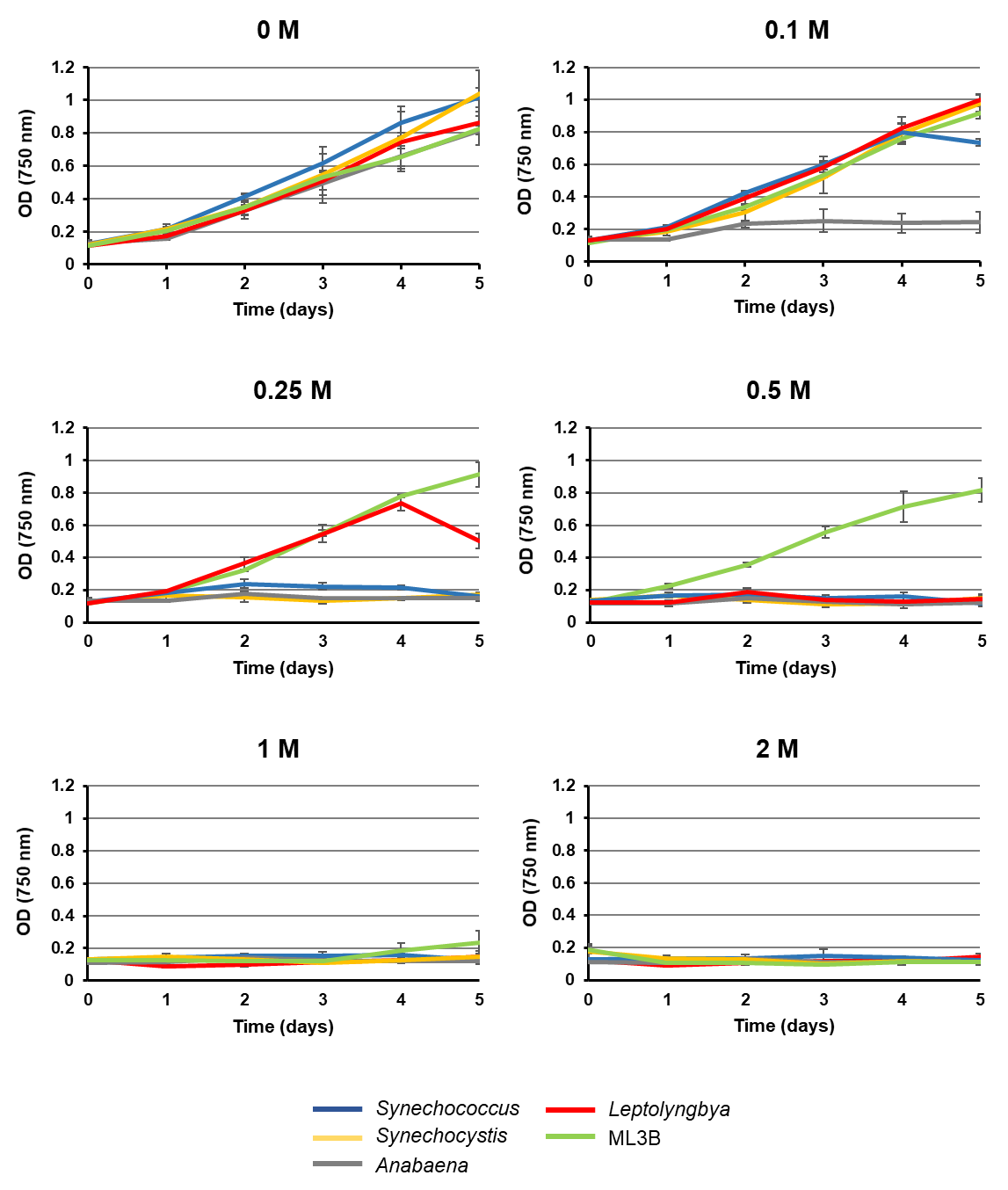


Figure S5. Growth tolerance of strains in high sodium bicarbonate media. Strains were grown in BG‑11 medium supplemented with sodium bicarbonate (NaHCO_3_) at concentrations ranging from 0 to 2 M. Cultures were grown for 5 days and the optical density (OD) was measured at 750 nm each day. Error bars represent standard deviations from biological triplicates.

**Table S1.** List of strains and plasmids.

| **Strain** | **Description** | **Reference** |
| --- | --- | --- |
| ***E. coli* strains** |  |  |
| DH10B | Cloning strain | Gibco BRL |
| AM1359 | Conjugal donor strain, Ap^r^, Tc^r^, Cm^r^ | Yoon & Golden, 1998 |
| AM5501 | DH10B strain harboring helper plasmid pAM5505 and conjugal plasmid pRL443, Ap^r^, Tc^r^, Cm^r^ | Bishé *et al.*, 2019 |
| WM3064 | DAP auxotroph strain of E. coli carrying the RP4 conjugal machinery on its chromosome | Wetmore et al 2015 |
| AM6013 | WM3064 carrying pRL623 | This study |
| DH5α | Cloning strain | Laboratory collection |
| DB3.1 | Cloning strain | Laboratory collection |
| One Shot TOP10 | Cloning strain | Invitrogen |
| **Cyanobacterial strains** | |  |
| *Synechococcus elongatus* PCC 7942 | Wild type | Laboratory collection |
| *Synechococcus elongatus* PCC 7942 ∆*ago* | Strain AMC2664, marker less deletion of the Argonaute (*ago*) gene (Synpcc7942_1534) | Taton et al., 2023 |
| *Synechocystis* sp. PCC 6803 | Wild type | Laboratory collection |
| *Anabaena* sp. PCC 7120 | Wild type | Laboratory collection |
| *Leptolyngbya* BL0902 | Wild type | Taton *et al.*, 2012 |
| ML2-A | Cyanobacterial strain isolated from Mono Lake, CA | This study |
| ML2-C1 | Cyanobacterial strain isolated from Mono Lake, CA | This study |
| ML2-C2 | Cyanobacterial strain isolated from Mono Lake, CA | This study |
| ML3-B | Cyanobacterial strain isolated from Mono Lake, CA | This study |
| **Plasmids** |  |  |
| pAM4818 | CYANO-VECTOR plasmid containing *aadA* (Sp/Sm resistance), Sp^r^, Sm^r^ | Taton et al., 2014 |
| pAM4819 | CYANO-VECTOR plasmid containing *aphI* (Km/Nm resistance), Km^r^, Nm^r^ | Taton et al., 2014 |
| pAM4836 | CYANO-VECTOR plasmid containing chromosomal integration site NS1 for *Synechococcus elongatus* PCC 7942, Ap^r^, Tc^r^ | Taton et al., 2014 |
| pAM4860 | CYANO-VECTOR plasmid containing promoter-reporter device (PconII driving *yfp*), Ap^r^ | Taton et al., 2014 |
| pAM5068 | RSF1010Y25F-*aphI*-*ccdB*/SwaI, Km^r^, Nm^r^ | Taton et al., 2014 |
| pAM5105 | CYANO-VECTOR plasmid containing *ccdB*-RiboJ-oRBS-*yfp* | Taton et al., 2014 |
|  |  |  |
|  |  |  |
| pAM5203 | NS1TC-*aadA*-*ccdB*-RiboJ-oRBS-*yfp*, Sp^r,^ Sm^r^ | This study |
| pAM6017 | NS1TC-*aadA*-PconII-oRBS-*yfp*, Sp^r,^ Sm^r^ | This study |
| pAM5263 | RSF1010Y25F-*aphI* | This study |
| pAM5264 | RSF1010Y25F-*aphI-PconII-oRBS-yfp* | This study |
| pAM5329 | NS1-*aadA* | Taton et al., 2017 |
| pAM5404 | RSF1010-mobAY25F-RK2*bom*, Ap^r^ | Bishé *et al.*, 2019 |
| pAM5409 | RSF1010-*mob*AY25F-RK2*bom*-*aadA*-PconII-oRBS-*yfp*, Sp^r^, Sm^r^ | Bishé *et al.*, 2019 |
| pAM5612 | RSF1010-Y25F-*aphI*-PconII-oRBS-*yfp*, Km^r^, Nm^r^ | This study |
| pAM5704-pAM5728 | RSF1010Y25F-*aphI*-PconII*(Se01-Se25)-oRBS-*yfp* | This study |
| pAM5729 | RSF1010Y25F-*aphI*-oRBS-*yfp* | This study |
| pRL443 | Conjugal plasmid, derivative of RP4, Ap^r^ | Elhai *et al.*, 1997 |
| pRL623 | Helper plasmid carrying Mob, ColK and methylase genes M.AvaI, M.Eco47II, M.EcoT22I, Cm^r^ | Elhai *et al.*, 1997 |

**Table** S**2**. List of primers

| Primer Name | Primer Sequence | Reference |
| --- | --- | --- |
| conII-REF-F | CTTGACAATTAATCATCGGCTCGTATAATGGTACCCATTT | This study |
| conII-REF-R | AAATGGGTACCATTATACGAGCCGATGATTAATTGTCAAGACGT | This study |
| conII-n5-F_a | CTTGACAATTAATCATCGGCNNGNNTAANGGTACCCATTT | This study |
| conII-n5-R_a | AAATGGGTACCNTTANNCNNGCCGATGATTAATTGTCAAGACGT | This study |
| conII-n5-F_b | CTTGACAATTAATCATCGGCNNGTANNNTGGTACCCATTT | This study |
| conII-n5-R_b | AAATGGGTACCANNNTACNNGCCGATGATTAATTGTCAAGACGTC | This study |
| PLIB-01F | GGCCAATAACCCAGGGATTTCCGGTTTCGAATTGAGATTGACG | This study |
| PLIB-01R | GCCGGGGAGCTCCTTCATTTTCCTGCAGGGCCGAGTTTGTAC | This study |
| aphi_seq_FW | ATGGCAAGATCCTGGTATCG | This study |
| aphi_seq_RV | TTATGCCTCTTCCGACCATC | This study |
| aadA_seq_FWD | TACTGCGCTGTACCAAATGC | This study |
| aadA_seq_REV | TGATTTGCTGGTTACGGTGA | This study |
| YFP_RSF_sq_FW | CCTGAAGTTCATCTGCACCA | This study |
| YFP_RSF_sq_RV | GAACTCCAGCAGGACCATGT | This study |
| pconii_seq_fw | GATGGTCGGAAGAGGCATAA | This study |
| pconii_seq_rv | GAACTTCAGGGTCAGCTTGC | This study |
| 16S27F | AGAGTTTGATCCTGGCTCAG | Taton et al., 2003 |
| 23S30R | CTTCGCCTCTGTGTGCCTAGGT | Taton et al., 2003 |
| 16S378F | GGGGAATTTTCCGCAATGGG | Taton et al., 2003 |
| 16S784R | GGACTACWGGGGTATCTAATCCC | Taton et al., 2003 |

Table S3. BLASTN hits for bioprospected strains. Sequences were obtained by querying the 16S rRNA gene sequences in the GenBank database. Redundant, closely related clones obtained from the same source were removed.

| Description* | Accession | Query Coverage | Score | E-value | Identity | Origin of strain |
| --- | --- | --- | --- | --- | --- | --- |
| **ML2A** |  |  |  |  |  |  |
| Uncultured bacterium clone GBII-87 | GQ441350.1 | 100% | 2348 | 0 | 98.07% | The Netherlands |
| Uncultured bacterium clone T-05_10 | KP793940.1 | 100% | 2337 | 0 | 97.92% | El Tatio, Chile |
| *Leptolyngbya* sp. 0BB30S02 | AJ639892.1 | 100% | 2324 | 0 | 97.77% | - |
| *Leptolyngbya* sp. LEGE 07080 | HM217085.1 | 100% | 2302 | 0 | 97.47% | Porto, Portugal |
| *Leptolyngbya* antarctica ANT.LAC.1 | AY493588.1 | 99% | 2298 | 0 | 97.54% | Antarctica |
| **ML2-C1** |  |  |  |  |  |  |
| Uncultured bacterium clone GBII-15 | [GQ441289.1](https://www.ncbi.nlm.nih.gov/nucleotide/GQ441289.1?report=genbank&log$=nucltop&blast_rank=1&RID=NGKGRSKX016) | 100% | 2206 | 0 | 98.33% | The Netherlands |
| Uncultured cyanobacterium clone T-05_10 | KP793940.1 | 100% | 2200 | 0 | 98.25% | El Tatio, Chile |
| *Leptolyngbya* antarctica ANT.LAC.1 | AY493588.1 | 99% | 2165 | 0 | 97.85% | Antarctica |
| Nodosilinea nodulosa LEGE 06104 | KU569325.1 | 100% | 2161 | 0 | 97.69% | - |
| Nodosilinea sp. ACSSI 330 | MT425944.1 | 100% | 2159 | 0 | 97.69% | - |
| **ML2-C2** |  |  |  |  |  |  |
| Uncultured bacterium clone GBII-15 | [GQ441289.1](https://www.ncbi.nlm.nih.gov/nucleotide/GQ441289.1?report=genbank&log$=nucltop&blast_rank=1&RID=NGKGRSKX016) | 100% | 2222 | 0 | 99.59% | The Netherlands |
| Uncultured cyanobacterium clone T-05_10 | KP793940.1 | 100% | 2217 | 0 | 99.51% | El Tatio, Chile |
| Uncultured cyanobacterium clone R8-R56 | DQ181691.1 | 99% | 2182 | 0 | 99.09% | Antarctica |
| *Leptolyngbya* antarctica ANT.LAC.1 | AY493588.1 | 99% | 2180 | 0 | 99.09% | Antarctica |
| *Leptolyngbya* sp. B-CY-NM2 | KX086280.1 | 96% | 2150 | 0 | 99.74% | Turkey |
| **ML3B** |  |  |  |  |  |  |
| Nodosilinea sp. CENA523 | KF246490.1 | 99% | 2475 | 0 | 99.13% | Pantanal wetlands, Brazil |
| *Leptolyngbya* sp. KIOST-1 | [JX401929.1](https://www.ncbi.nlm.nih.gov/nucleotide/JX401929.1?report=genbank&log$=nucltop&blast_rank=4&RID=N0V2VADN01R) | 100% | 2438 | 0 | 99.48% | Ansan, South Korea |
| *Leptolyngbya* sp. 0BB32S02 | [AJ639894.1](https://www.ncbi.nlm.nih.gov/nucleotide/AJ639894.1?report=genbank&log$=nucltop&blast_rank=6&RID=N0V2VADN01R) | 100% | 2351 | 0 | 97.32% | - |
| Nodosilinea cf. nodulosa LEGE 10377 | [JQ927349.1](https://www.ncbi.nlm.nih.gov/nucleotide/JQ927349.1?report=genbank&log$=nucltop&blast_rank=8&RID=N0V2VADN01R) | 99% | 2337 | 0 | 97.24% | Coastal Portugal |
| Uncultured bacterium clone BJGMM-3s-409 | [JQ801062.1](https://www.ncbi.nlm.nih.gov/nucleotide/JQ801062.1?report=genbank&log$=nucltop&blast_rank=11&RID=N0V2VADN01R) | 100% | 2329 | 0 | 97.04% | Yellow River, China |

**Table S4. BLASTN sequence queries for bioprospected strains in FASTA format.**

>ML2A

GCTCAGGATGAACGCTGGCGGCGTGCTTAACACATGCAAGTCGAACGGACCCTTCGGGGTTAGTGGCGGACGGGTGAGTAACGCGTGAGGATCTGCCCTTAGGAGGGGGACAACAGTTGGAAACGACTGCTAATACCCCATATGCCGAGAGGTGAAATGTAATTCGCCTGAGGATGAACTCGCGTCTGATTAGCTAGTTGGTGGTGTAAGGGACCACCAAGGCGACGATCAGTAGCTGGTCTAAGAGGATGATCAGCCACACTGGGACTGAGACACGGCCCAGACTCCTACGGGAGGCAGCAGTGGGGAATTTTCCGCAATGGGCGAAAGCCTGACGGAGCAACGCCGCGTGAGGGAGGAAGGCCTTAGGGTTGTAAACCTCTTTTCTCTGGGAAGAAGAACTGACGGTACCAGAGGAATAAGCCTCGGCTAACTCCGTGCCAGCAGCCGCGGTAAGACGGAGGAGGCAAGCGTTATCCGGAATTATTGGGCGTAAAGCGTCCGCAGGCGGTTTTTTAAGTCTGTTGTCAAAGCCCACAGCTCAACTGTGGATCGGCAATGGAAACTGGGAGACTAGAGTGTGGTAGGGGTAGAGGGAATTCCCGGTGTAGCGGTGAAATGCGTAGATATCGGGAAGAACACCAGTGGCGAAGGCGCTCTACTGGGCCACAACTGACGCTGAGGGACGAAAGCTAGGGGAGCGAAAGGGATTAGATACCCCTGTAGTCCTAGCTGTAAACGATGGATACTAGGTGTTGGACGTATCGACCCGTGCAGTACCGTAGCTAACGCGTTAAGTATCCCGCCTGGGGAGTACGCACGCAAGTGTGAAACTCAAAGGAATTGACGGGGGCCCGCACAAGCGGTGGAGGATGTGGTTTAATTCGATGCAACGCGAAGAACCTTACCAAGGCTTGACATGTCGCGAATCTTTGCGAGAGCAGAGAGTGCCTTCGGGAGCGCGAACACAGGTGGTGCATGGCTGTCGTCAGCTCGTGTCGTGAGATGTTGGGTTAAGTCCCGCAACGAGCGCAACCCACGTCTTTAGTTGCCAGCATTAAGTTGGGCACTCTAGAGAGACTGCCGGGGACAACTCGGAGGAAGGTGTGGACGACGTCAAGTCATCATGCCCCTTACGTCTTGGGCTACACACGTCCTACAATGCTACGGACAGAGGGCAGCAAGCCAGCGATGGTTAGCAAATCCCATAAACCGTGGCTCAGTTCANATTGCAGGCTGCAACTCGCCTGCATGAAGGNGGAATCGCTAGTAATCGCNNTCAGCATACTGCGGTGAATACGTTTCCCGGGCCTTGTACACACCGCCCGTCACACCATGGGAGTT

>ML2-C1

TCAGGATGAACGCTGGCGGCGTGCTTAACACATGCAAGTCGAACGGACCCTTCGGGGTTAGTGGCGGACGGGTGAGTAACGCGTGAGGATCTGCCCTTAGGAGGGGAACAACAGTTGGAAACGACTGCTAATGCCCCATATGCCGAGAGGTGAAACGGTTAAATCCGCCTGAGGATGAACTCGCGTCTGATTAGCTAGTTGGTGGGGTAAGGGCCCACCAAGGCGACGATCAGTAGCTGGTCTAAGAGGATGATCAGCCACACTGGGACTGAGACACGGCCCAGACTCCTACGGGAGGCAGCAGTGGGGAATTTTCCGCAATGGGCGCAAGCCTGACGGAGCAACGCCGCGTGAGGGAGGAAGGCCTTAGGGTTGTAAACCTCTTTTCTCTGGGAAGAAGAACTGACGGTACCAGAGGAATAAGCCTCGGCTAACTCCGTGCCAGCAGCCGCGGTAAGACGGAGGAGGCAAGCGTTATCCGGAATTATTGGGCGTAAAGCGTCCGTAGGCGGTTTCTTAAGTCTGTTGTCAAAGGTCACAGCTCAACTGTGGATCGGCAATGGAAACTGGGGAACTTGAGTGTGGTAGGGGTAGAGGGAATTCCCGGTGTAGCGGTGAAATGCGTAGATATCGGGAAGAACACCAGTGGCGAAGGCGCTCTACTGGGCCACAACTGACGCTGATGGACGAAAGCTAGGGGAGCGAAAGGGATTAGATACCCCTGTAGTCCTAGCTGTAAACGATGGATACTAGGTGTTGGACGTATCGACCCGTGCAGTACCGTAGCTAACGCGTTAAGTATCCCGCCTGGGGAGTACGCACGCAAGTGTGAAACTCAAAGGAATTGACGGGGGCCCGCACAAGCGGTGGAGGATGTGGTTTAATTCGATGCAACGCGAAGAACCTTACCAAGGCTTGACATGTCACGAATCCTTCAGAGATGAGGGAGTGCCTTCGGGAGCGTGAACACAGGTGGTGCATGGCTGTCGTCAGCTCGTGTCGTGAGATGTTGGGTTAAGTCCCGCAACGAGCGCAACCCACGTTTTTAGTTGCCAGCATTAAGTTGGGCACTCTAGAGAGACTGCCGTGGACAACACGGAGGAAGGTGTGGACGACGTCAAGTCATCATGCCCCTTACGCCTTGGGCTACACACGTCCTACAATGTTACAGACAGAGGGCAGCAAGCCAGCGATGGTTAGCAAATCCCATAAACTGTGGCTCAGTTCAGATTGCAGGCTGCAACTCGCCTGCATGAA

>ML2-C2

CTCAGGATGAACGCTGGCGGCGTGCTTAACACATGCAAGTCGAACGGACCCTTCGGGGTTAGTGGCGGACGGGTGAGTAACGCGTGAGGATCTGCCCTTAGGAGGGGAACAACAGTTGGAAACGACTGCTAATGCCCCATATGCCGAGAGGTGAAACGGTTAATTCCGCCTGAGGATGAACTCGCGTCTGATTAGCTAGTTGGTGGGGTAATAGCCCACCAAGGCGACGATCAGTAGCTGGTCTAAGAGGATGATCAGCCACACTGGGACTGAGACACGGCCCAGACTCCTACGGGAGGCAGCAGTGGGGAATTTTCCGCAATGGGCGAAAGCCTGACGGAGCAACGCCGCGTGAGGGAGGAAGGCCTTAGGGTTGTAAACCTCTTTTCTCTGGGAAGAAGAACTGACGGTACCAGAGGAATAAGCCTCGGCTAACTCCGTGCCAGCAGCCGCGGTAAGACGGAGGAGGCAAGCGTTATCCGGAATTATTGGGCGTAAAGCGTCCGTAGGCGGCTTTTTAAGTCTGTTGTCAAAGCCCACAGCTCAACTGTGGATCGGCAATGGAAACTGGGGAGCTTGAGTGTGGTAGGGGTAGAGGGAATTCCCGGTGTAGCGGTGAAATGCGTAGATATCGGGAAGAACACCAGTGGCGAAGGCGCTCTACTGGGCCACAACTGACGCTGATGGACGAAAGCTAGGGGAGCGAAAGGGATTAGATACCCCTGTAGTCCTAGCTGTAAACGATGGATACTAGGTGTTGGACGTATCGACCCGTGCAGTACCGTAGCTAACGCGTTAAGTATCCCGCCTGGGGAGTACGCACGCAAGTGTGAAACTCAAAGGAATTGACGGGGGCCCGCACAAGCGGTGGAGGATGTGGTTTAATTCGATGCAACGCGAAGAACCTTACCAAGGCTTGACATGTCGCGAATCTTTGCGAGAGCAGAGAGTGCCTTCGGGAGCGCGAACACAGGTGGTGCATGGCTGTCGTCAGCTCGTGTCGTGAGATGTTGGGTTAAGTCCCGCAACGAGCGCAACCCACGTTTTTAGTTGCCAGCATTAAGTTGGGCACTCTAGAGAGACTGCCGTGGACAACACGGAGGAAGGTGTGGACGACGTCAAGTCATCATGCCCCTTACGTCTTGGGCTACACACGTCCTACAATGCTTCGGACAGAGGGCAGCAAGCCAGCGATGGTTAGCAAATCCCATAAACCGA

>ML3B

GGCTCAGGATGAACGCTGGCGGCGTGCTTAACACATGCAAGTCGAACGGACCCTTCGGGGTTAGTGGCGGACGGGTGAGTAACGCGTGAGGATCTGCCCTTAGGATGGGGACAACCACTGGAAACGGTGGCTAATACCCAATGTGCCGAGAGGTGAAACATTTATGGCCTGAGGATGAACTCGCGTCTGATTAGCTAGTTGGTGAGGTAAGGGCTCACCAAGGCGACGATCAGTAGCTGGTCTAAGAGGATGATCAGCCACACTGGGACTGAGACACGGCCCAGACTCCTACGGGAGGCAGCAGTGGGGAATTTTCCGCAATGGGCGCAAGCCTGACGGAGCAACGCCGCGTGAGGGAGGAAGGCCTTAGGGTTGTAAACCTCTTTTCTCTGGGAAGAAGTTCTGACGGTACCAGAGGAATAAGCCTCGGCTAACTCCGTGCCAGCAGCCGCGGTAAGACGGAGGAGGCAAGCGTTATCCGGAATTATTGGGCGTAAAGCGTCCGCAGGCGGTTTATTAAGTCTGTTGTCAAAGGTCACAGCTCAACTGTGGATCGGCAATGGAAACTGGTGAACTTGAGTGTGGTAGGGGTAGAGGGAATTCCCGGTGTAGCGGTGAAATGCGTAGATATCGGGAAGAACACCAGTGGCGAAGGCGCTCTACTGGGCCACAACTGACGCTGAGGGACGAAAGCTAGGGGAGCGAAAGGGATTAGATACCCCTGTAGTCCTAGCTGTAAACGATGGATACTAGGTGTTGGACGTATCGACCCGTGCAGTACCGTAGCTAACGCGTTAAGTATCCCGCCTGGGGAGTACGCACGCAAGTGTGAAACTCAAAGGAATTGACGGGGGCCCGCACAAGCGGTGGAGGATGTGGTTTAATTCGATGCAACGCGAAGAACCTTACCAAGGCTTGACATGTCGCGAATCTCTGAGAGATCAGAGAGTGCCTTCGGGAGCGCGAACACAGGTGGTGCATGGCTGTCGTCAGCTCGTGTCGTGAGATGTTGGGTTAAGTCCCGCAACGAGCGCAACCCACGTTTTTAGTTGCCAGCATTAAGTTGGGCACTCTAAAGAGACTGCCGGGGACAACTCGGAGGAAGGTGTGGACGACGTCAAGTCATCATGCCCCTTACGTCTTGGGCTACACACGTCCTACAATGCTACAGACAGAGGGCAGCGAGCGCGCGAGTGCAAGCAAATCCCATAAACTGTGGCTCAGTTCAGATTGCAGGCTGCAACTCGCCTGCATGAANGNGGAATCGCTAGTAATCGCCNGGTCAGCANNACGGCGGTGAATACGTTCCCGGGCCTTGTACACCACCGCCCGTCACACCATGGGGAGTTNGGCCACNCCCGAAGTCGTTACTCTAACCGTTC
